# Is there symmetry in motor imagery? Exploring different versions of the mental chronometry paradigm

**DOI:** 10.3758/s13414-016-1112-9

**Published:** 2016-05-12

**Authors:** Stephan F. Dahm, Martina Rieger

**Affiliations:** Institute of Psychology, UMIT - University for Health Sciences Medical Informatics and Technology, Hall in Tyrol, Austria

**Keywords:** Motor imagery, Mental chronometry, Bimanual coordination, Coordination constraints, Cyclical movements, Psychometric functions

## Abstract

Motor imagery and motor execution share similar processes. However, only some factors that affect motor execution affect motor imagery in the same way. We investigated whether bimanual coordination constraints (parallel movements are performed slower than symmetric movements) are observed in motor imagery and whether the way of implementing the mental chronometry paradigm, which is used to investigate motor imagery, influences the results. Participants imagined and executed repetitive symmetric and parallel bimanual movements in three different tasks. Participants performed a certain number of movement repetitions (number task), repeated movements for a fixed duration (duration task), and performed movements in synchrony with pacing sounds (synchronization task). In both, imagination and execution, inter-response intervals were longer with parallel movements than with symmetric movements (number task and duration task), and the percentage of correct movements was lower with parallel than with symmetric movements (synchronization task). Performance of imagined and executed movements was correlated in all tasks. However, imagination took longer or was rated as less accurate than execution, and in the synchronization task the coordination constraint affected accuracy more in execution than in imagination. Thus, motor imagery and overt execution involve shared and unique processes. The synchronization task offers a promising alternative to investigate motor imagery, because the speed-accuracy trade-off is taken into account, different tempi can be used, and psychometric functions can be calculated.

**Electronic supplementary material:**

The online version of this article (doi:10.3758/s13414-016-1112-9) contains supplementary material, which is available to authorized users.

## Introduction

Motor imagery designates the mental simulation of movements without actual execution (Jeannerod, 1995). It is assumed that imagination and execution are functionally equivalent, i.e., that they rely on similar processes (Jeannerod, 1995). Nevertheless, differences between imagination and execution are sometimes observed (Decety, Jeannerod, & Prablanc, 1989; Cerritelli, Maruff, Wilson, & Currie, 2000). In particular, it remains unclear which factors that affect motor execution also affect motor imagery and under which circumstances they do so. The first goal of the present study therefore was to investigate whether bimanual coordination constraints (symmetric movements are performed faster than parallel movements) are observed in motor imagery. This was done using the mental chronometry paradigm, in which durations of imagined and executed movements are compared (Jeannerod, 1994). There are several ways to implement the mental chronometry paradigm, which, to the best of our knowledge, have not been systematically compared. Therefore, the second goal of this study was to investigate the impact of different versions of the mental chronometry paradigm on the presence of bimanual coordination constraints in motor imagery.

The assumption of similar processes during imagination and execution has been supported by studies using functional brain imaging techniques, which show similar brain activity in imagination and execution (Hanakawa et al., 2003; Lorey et al., 2013; Lotze et al., 1999), and electrophysiological studies, which show similar oscillatory power changes in imagination and execution (Hermes et al., 2011; Schnitzler, Salenius, Salmelin, Jousmäki & Hari, 1997). Further support for the assumption of functional equivalence of imagination and execution comes from studies using the mental chronometry paradigm. In many instances, the durations of imagined and executed movements are similar and they are correlated (for an overview see Guillot & Collet, 2005). Furthermore, imagination and execution often show a similar pattern across experimental conditions (Dahm & Rieger, 2016; Decety & Michel, 1989; Papaxanthis, Schieppati, Gentili, & Pozzo, 2002; Papaxanthis, Pozzo, Kasprinski, & Berthoz, 2003; Frak, Paulignan, & Jeannerod, 2000; Wilson, Maruff, Ives & Currie, 2001). For instance, durations of arm movements increase with added weight, both in imagination and execution (Papaxanthis et al., 2002), the difficulty of end-positions influences durations of imagined and executed arm movements (Brinkman, Stolk, Dijkerman, de Lange & Toni, 2014; Frak, Paulignan & Jeannerod, 2000) and in bimanual coordination durations are higher with dissimilar than similar targets, both in imagination and execution (Dahm & Rieger, 2016).

Nevertheless, motor imagery and motor execution differ in some aspects. In motor imagery, the neural innervation of the muscles needs to be inhibited so that no actual movement occurs (Guillot, Di Rienzo, MacIntyre, Moran & Collet, 2012) . Consequently, feedback about the consequences and progress of the movement is not available. Probably due to these differences, some factors that influence motor execution are not completely taken into account during motor imagery (for an overview see Guillot, Hoyek, Louis & Collet, 2012). For instance, unfamiliar movements, such as movements to awkward and uncommon postures (Parsons, 1994) or typing in a different style than usual (Rieger, 2012), are not adequately represented in motor imagery. Some factors that influence execution even have a stronger influence on motor imagery. For instance, walking with added weight has a stronger influence on imagination durations than on execution durations. This probably occurs because another factor influencing movement duration—the exertion of muscle forces compensating for added weight—may be neglected in motor imagery (Decety et al., 1989; Cerritelli et al., 2000; Wilson et al., 2001). Similarly, slower performance of the nondominant hand compared with the dominant hand is more pronounced in imagination than in execution (Maruff et al., 1999). Altogether, the results suggest that even though in most instances factors that constrain motor execution similarly constrain motor imagery, this may not always be the case or they may constrain motor imagery to a lesser degree. The first goal of the present study was to investigate to what extent coordination constraints of bimanual movements affect motor imagery.

Bimanual movements are performed with both hands. The hands can be coordinated in different ways. Symmetric movements are mirrored along the body midline (e.g., both hands move toward the body midline at the same time). Parallel movements, which are a specific form of asymmetric movements, are performed in the same direction in external space (e.g., both hands move to the left at the same time). Symmetric movements are easier to perform than asymmetric or parallel movements (Spijkers, Heuer, Kleinsorge, & van der Loo, 1997; Swinnen, Dounskaia, Walter & Serrien, 1997). People even switch into symmetry when executing parallel movements, but usually not vice versa (Mechsner, Kerzel, Knoblich, & Prinz, 2001). This symmetry tendency is due to a “coalition of constraints” (Swinnen & Wenderoth, 2002), present at the motor level (e.g., due to neuronal crosstalk of motor commands between the hemispheres; Cardoso de Oliveira, 2002; Swinnen et al., 1997) and at the perceptual-cognitive level (Mechsner et al., 2001).

Bimanual coordination constraints at the motor level (Heuer, Spijkers, Kleinsorge, & van der Loo, 1998) and at the perceptual-cognitive level (Dahm & Rieger, 2016) both seem to influence motor imagery. However, the effect of bimanual constraints seems to be weaker in imagery than in execution (Heuer et al., 1998). Heuer and colleagues (1998, Exp. 2) asked participants to synchronize bimanual movements with pacing sounds. Participants always executed long amplitudes with one hand. With the other hand, they executed or imagined either amplitudes of the same length (long) or alternated between short and long amplitudes. Imagery was supported by bars on a computer monitor that were increasing and decreasing in correspondence with the movement amplitude and the pacing sounds. Alternating between short and long amplitudes with one hand influenced the amplitudes of the other hand, which was supposed to always move the same amplitude. This was the case both in imagination and execution, but the effect was stronger in execution than in imagination.

In the present study, we investigated whether bimanual coordination constraints (symmetric movements are easier than parallel movements) are observed in motor imagery of fast cyclic movements. In contrast to Heuer and colleagues (1998) and similar to Dahm and Rieger (2016), movements of both arms were imagined. In contrast to Dahm and Rieger (2016), repetitive movements were investigated. Importantly, no external imagery support (i.e., visual signals on the monitor) was given. In the study by Heuer and colleagues (1998), not only imagery, but also the visual signals on the monitor, may have influenced the amplitudes of the hand which was supposed to move constant amplitudes (Kilner, Hamilton & Blakemore, 2007). In the present study, participants performed repetitive bimanual movements on the horizontal axis. Movements were either symmetric or parallel and either imagined or executed. Based on previous findings (Dahm & Rieger, 2016; Heuer et al., 1998), we expected an influence of bimanual coordination constraints on motor imagery durations. However, this influence might be stronger in motor execution than in motor imagery (Heuer et al., 1998). Furthermore, we expected that individual differences in motor execution are reflected in motor imagery, resulting in significant correlations between execution and imagination.

In the present study, participants were explicitly asked to imagine the movements. In explicit motor imagery tasks, it is possible to obtain objective performance measures if the movement is partly imagined and partly executed (Heuer et al., 1998, see above). If the movement is fully imagined, most studies assess movement duration using predetermined start and end points of the movement (e.g., walking from one location to another). Participants usually indicate start and/or end of the movement by pressing a key/stopwatch with the movement irrelevant hand (Bakker et al., 2007; Chabeauti et al., 2012; Cerritelli et al., 2000; Decety & Jeannerod, 1996; Grealy & Shearer, 2008; Papaxanthis et al., 2002), by telling the experimenter to press a key/stopwatch (Munzert, 2002; Rieger & Massen, 2014; Wilson et al., 2001) or by performing the first and last part of the movement (Caeyenberghs et al., 2009; Dahm & Rieger, 2016; Rieger, 2012). However, there are alternative ways to implement the mental chronometry paradigm. Rather than asking participants to perform a movement up to a predetermined point, participants may be asked to perform the movement for a specific duration. They can then be asked to report the progress of the movement. For example, participants may be asked to walk mentally into a certain direction. Then, after a specific duration, they are asked to stop and to report their current position or number of steps they have taken. A third way to measure motor imagery, at least for repetitive movements, is that participants are asked to imagine their movements in synchrony with pacing sounds. Afterwards they rate the accuracy of their (imagined or executed) performance (Clark & Williamon, 2012, for the use of accuracy ratings in imagined musical performance). The advantages of the latter version are that movement speed/tempo and the number of repetitions can be manipulated by the experimenter and that it is possible to calculate psychometric functions due to the use of different tempi. However, a disadvantage may be that data collection takes longer to obtain reliable measurements at different tempi. In contrast, in the first two versions of the mental chronometry paradigm reliable data may be collected relatively shortly. However, at least when simple repetitive arm movements (back and forth) are investigated, the first two versions have the disadvantage that participants have to count their movements, which may be disruptive. Thus, to investigate motor imagery of repetitive movements, at least three different versions of the mental chronometry paradigm can be thought of. The second goal of the present study was to investigate the impact of different versions of the mental chronometry paradigm on the presence of bimanual coordination constraints in motor imagery.

We compared the three outlined versions of the mental chronometry paradigm. In the number task, which is based on the most common version of the mental chronometry paradigm, we asked participants to imagine or execute bimanual movements a certain number of times and measured movement duration. In the duration task, we asked participants to imagine or execute movements for a fixed duration and afterwards asked them to report the number of movements they had performed. In the synchronization task, participants were asked to synchronize their imagined or executed movements with pacing sounds. This was done in different tempi. They were then asked to rate the percentage of correct, synchronously performed movements. We expected better performance (i.e., shorter durations or higher accuracy) in symmetric than parallel movements in all three versions of the mental chronometry paradigm. Because the number and the duration task are very similar, we expected no differences between them. In the synchronization task, we expected that movement accuracy decreases with increasing tempo and that at extreme tempi (very fast or very slow) performance differences between symmetric and parallel movements become weaker or disappear (floor and ceiling effects). Furthermore, we expected steeper slopes in parallel than in symmetric movements. The steepness of the slope indicates how much movement accuracy depends on tempo. In all tasks, we compared actual execution with reported execution to investigate whether the reported data reflect actual performance.

## Methods

### Participants

Originally 24 students participated in this study. Three participants were excluded from analysis. One participant reported not to have been able to concentrate during the experiment, and two participants showed no speed-accuracy trade-off in reported performance in the synchronization task, indicating that they may not have performed the task as instructed. The remaining 21 participants (19 females) were on average 25.4 years old (*SD* = 6.3 years), and all were right-handed (laterality index between 50 and 100) as assessed with the Edinburgh Inventory (Oldfield, 1971). They were paid 9 Euros/hour for their participation or received course credit. The experiment lasted approximately 2 hours and was approved by the local ethics committee. All participants gave informed consent.

The sample size was calculated using G*Power 3.1.6. It was based on a repeated-measures ANOVA design. Number of factors was set at two, considering coordination pattern and action. For coordination pattern, we assumed a medium effect size (*f* = .25). We further assumed high correlations between the four conditions (*r* = .75). Statistical significance was set at *p* < .05. Power (1-beta) was set at 0.9. This resulted in a sample size estimation of *N* = 24.

### Material and procedure

**Fig. 1:**
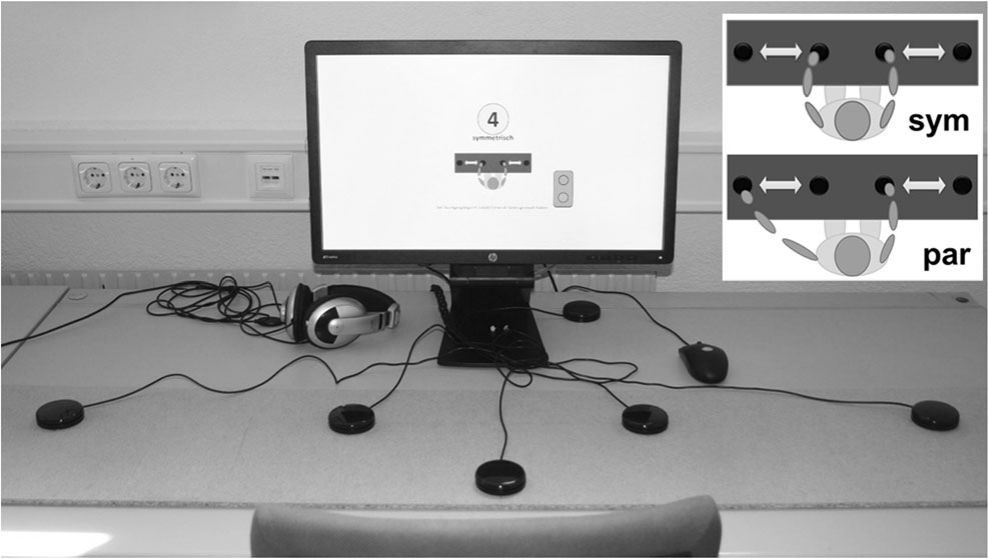
Stimuli on the screen and arrangement of response buttons. In the upper right corner, the first bimanual button press of symmetric (sym) and parallel (par) movements is depicted

Instructions were presented on an HPz23i monitor (screen resolution: 1920x1080). A board with five buttons (radius 3 cm) was placed on a table (Fig. 1). Four buttons were aligned horizontally in a row (distance between buttons: 40 cm; distance from the edge of the table: 30 cm). The two buttons on the left were used with the left hand. The two buttons on the right were used with the right hand. An additional button, located in the middle 15 cm closer to the body than the other buttons, was used in one of the tasks to indicate the completion of a trial by pressing it with the right hand (finish button). The experiment was programmed using the software Presentation^®^ (Version 17.1, www.neurobs.com).

All instructions were given in written form on the computer. An experimenter was present the entire time to answer any questions and to check whether participants followed the instructions. At the beginning of the experiment, participants received instructions that explained the symmetric and parallel movements. With symmetric movements, participants were asked to start by pressing the inner buttons with each hand and then to alternate between pressing the outer and inner buttons. With parallel movements, participants were asked to start by pressing the left buttons with each hand and then to alternate between the right and the left buttons (Fig. 1). In the execution conditions, (EXE) participants performed the movement. In the imagination conditions (IMA), they were asked to imagine how it feels to perform the movements and button presses.

Participants performed three different tasks: the number task, the duration task, and the synchronization task (for details see below). In every task, the main instructions started with two short clips illustrating six repetitive (symmetric or parallel) arm movements. Concurrent with the button presses either increasing numbers from 1 to 6, which represented counting (number task and duration task), or pacing sounds (synchronization task) were presented (see online supplemental material for video clips of the main instructions). Every task started with four practice trials, which were not included in the analysis. The order of tasks was counterbalanced across participants, resulting in six possible orders (each order was completed by 4 participants). The order of action conditions within tasks (imagination, execution) was blocked, counterbalanced across participants, and the same in all three tasks. After each action condition, single questions were used to assess participants’ concentration (from "very unconcentrated" to "very concentrated") and strength of representation of kinesthesis/touch (how it feels) and vision when performing the movement (from "not at all" to "very clear"). Participants gave their ratings by clicking with the computer mouse on a visual analogue scale (15.9 cm). The leftmost point was defined as 0 and the rightmost point as 100 (see supplemental material for an analysis of the data on strength of representation).

#### Number task

**Fig. 2:**
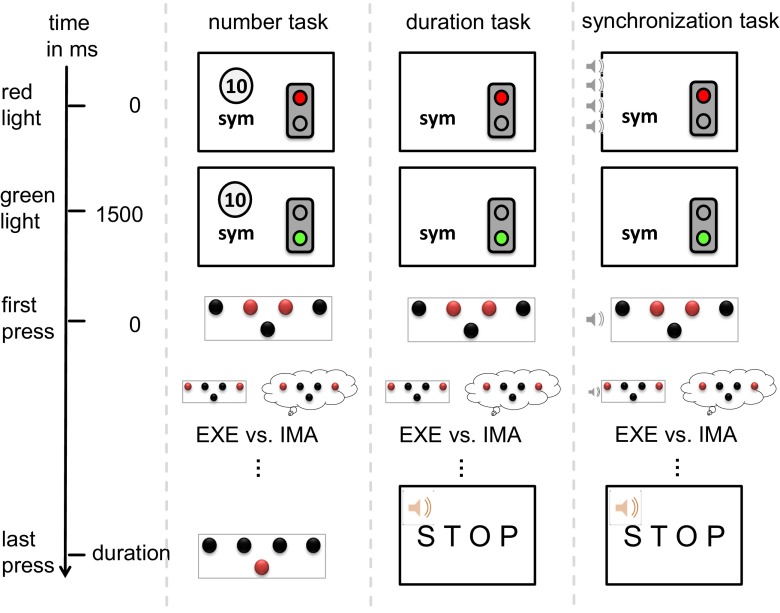
Trial procedures for the number task, the duration task, and the synchronization task. Instructions (accompanied by a red light) included information about the coordination pattern (symmetric, parallel), the requested number of repetitions (in the number task), and four sounds to indicate the following tempo (in the synchronization task). In all tasks, the red light went off and a green light below it turned on after 1500 ms. A trial started with the first bimanual press. Participants then executed (EXE) or imagined (IMA) the movements until the end of the trial. In the number task, participants pressed the finish button to indicate the end of a trial. In the duration task and the synchronization task, the end of a trial was indicated by a visual-acoustic signal, which was followed by questions concerning counts or perceived accuracy. Note that the depicted graphics are not scaled to the actual graphics on the screen

Participants were instructed to move as fast and accurate as possible for a predetermined number of button presses. They were asked to count their bimanual presses and told that pressing two buttons with both hands at the same time counted as one bimanual press. Before each trial, participants received instructions about the coordination pattern (symmetric or parallel) and the required number of movements. These instructions were accompanied by a red light (on the lower right side of the screen), which indicated that participants should read the instructions (Fig. 2). The red light turned green after 1500 ms. From then on, participants were able to start the trial when they felt ready. After the first button press, instructions were replaced with a blank screen. In IMA participants indicated the start of their imagination by pressing the first buttons and then imagined the remaining button presses. In both, imagination and execution, they indicated the completion of the required number of bimanual presses by pressing the finish button. The screen remained blank for 500 ms until the next trial started. Participants were asked to perform 5 different numbers of button presses (4, 8, 12, 16, and 20 presses). Each number of button presses was performed three times in each coordination pattern and each action condition resulting altogether in 60 trials. Within each action condition, numbers of button presses were randomized with the restriction that every number of button presses occurred once in each coordination pattern before a trial was repeated. The whole task took approximately 30 minutes.

#### Duration task

Participants were instructed to move as quickly and accurately as possible and were asked to count their bimanual presses. They were not informed about the duration of a trial in advance. Trial instructions informed about the coordination pattern and were accompanied by a red light as in the number task (Fig. 2). After the first button press, instructions were replaced with a blank screen. In IMA participants indicated the start of their imagination by pressing the first buttons and then imagined the remaining button presses. The end of a trial was signaled with the presentation of the word "STOP" (in capitalized black letters, font size 80) on an orange background and a concurrent deep tone (440 Hz, 1500 ms). At the end of each trial, participants were asked to report the number of bimanual presses that they had performed. Durations (3, 3.5, 4, 4.5, and 5 s) were presented three times in each coordination pattern and action condition resulting in altogether 60 trials. Within each action condition, durations were randomized with the restriction that every duration occurred once in each coordination pattern before a trial was repeated. The whole task took approximately 30 minutes.

#### Synchronization task

Participants were instructed to synchronize their movements to pacing sounds (1000 Hz, 10 ms) in different tempi. They were asked to perform a bimanual press concurrently with every sound. Before each trial, participants received instructions about the coordination pattern. This was accompanied by a red light as in the number task (Fig. 2). Trial instructions were further accompanied by four sounds indicating the subsequent tempo. After the first button press, instructions were replaced with a blank screen and the pacing sounds followed. In IMA participants indicated the start of their imagination by pressing the first buttons. They then imagined the remaining presses. After 20 sounds, the end of a trial was signaled with the presentation of the word "STOP" (capitalized black letters, font size 80) on an orange background and a concurrent deep tone (440 Hz, 1500 ms). Participants then reported the percentage of correct bimanual button presses (both hands in the correct coordination pattern synchronized with the sounds) on a visual analogue scale (15.9 cm, from 0% to 100%). Nine different tempi (360, 330, 300, 270, 240, 210, 180, 150, and 120 bpm), which resulted in the following inter-stimulus intervals (ISIs): 167, 182, 200, 222, 250, 286, 333, 400, and 500 ms, were presented. Each tempo was presented five times in each coordination pattern and action condition resulting altogether in 180 trials. Within each action condition, tempi were randomized with the restriction that every tempo occurred once in each coordination pattern before a trial was repeated. The whole task took approximately 40 minutes.

### Data analysis

In the number and duration tasks, we calculated the average inter-response interval (IRI): a) based on reports in the imagination condition; b) based on reports in the execution condition; and c) based on actual execution as a measure of performance. In the number task, IRIs for EXE were calculated using the duration between the first bimanual press and the last bimanual press divided by the number of requested bimanual presses minus one (because there is no preceding interval for the first button press). Note that we did not use the actual number of bimanual presses, because this is not possible in IMA (requested presses in EXE: *M* = 14; actual presses in EXE: *M* = 14.1, *SD =* 0.39). To calculate IRIs in IMA, we first calculated the average duration between the last bimanual button press and the press of the finish button in EXE. This duration was subtracted from the duration from start to finish in IMA. The resulting duration was divided by the number of requested bimanual presses minus one. The data from trials in which only four bimanual presses were requested were excluded from analysis, because they were less reliable (most likely due to the low number) than the data from other trials. Correlations between IRIs from trials with four presses and trials with more than four presses were significantly lower than correlations between IRIs from trials of more than four presses with each other (imagination: *r* = .71 and *r* = .94 respectively, *z* = 2.68; *p* = .007; execution: *r* = .66 and *r* = .96, respectively, *z* = 3.4; *p* < .001; mean correlations were calculated and compared using Fishers’ z-transformed values).

In the duration task, IRIs in EXE were calculated using the duration between the first and the last bimanual press divided by participants’ reported number of bimanual presses minus one (reported presses: *M* = 13.6, *SD =* 2.47; actual presses *M* = 13.8, *SD =* 2.6). To calculate IRIs in IMA, we first calculated the average difference between the duration of the first and last bimanual press and the requested duration in EXE. This value was added to the requested duration in IMA. The resulting duration was then divided by participants’ reported number of bimanual presses minus one.

In both, the duration and the number task, we additionally calculated the number of invalid button presses in execution and the actual IRIs of valid bimanual presses by using the durations between consecutive valid bimanual presses (data for the left and the right hand were averaged). A bimanual press was considered valid if both hands corresponded to the instructed pattern for two consecutive movements. Invalid button presses were not included in this analysis, because it was sometimes unclear which button presses represented one bimanual press when additional button presses were performed. For the main analysis, data were averaged over numbers of button presses (number task) and durations (duration task) (for detailed analyses depending on different numbers of button presses and durations see supplemental material).

In the synchronization task, we analyzed participants’ reported percentage of correct movements (COR%) in IMA and EXE. For EXE, we additionally calculated the actual COR%. Bimanual presses were considered valid if the coordination pattern corresponded to the instructions and both button presses belonged to the same sound (button presses belonged to the sound to which they were closest in time). The first four bimanual presses were not included into actual COR%. Furthermore, we calculated individual logistic regression functions for symmetric and parallel movements in IMA (COR%) and EXE (COR% and actual COR%). Based on these individual logistic regression functions, we compared the slopes (β) between conditions. For all tasks, we calculated the inter onset interval (IOI), i.e., the absolute value of the interval between button presses of the left and the right hand of valid bimanual presses as a measure of simultaneousness (see supplemental material for the analysis of IOI).

Dependent variables were analyzed using repeated-measures ANOVA. If Mauchly’s test indicated that the assumption of sphericity was violated, we report Huyn-Feld corrected degrees of freedom and *p* values. Further comparisons were conducted using *t* tests or additional analysis of variance with Sidak adjusted pairwise comparisons. Where appropriate, we report minimum (*p*_min_) or maximum (*p*_max_) statistical values. Statistical significance was set at *p* < 0.05. We compared a) executed and imagined performance based on reports and b) actual and reported performance in EXE separately. Because performing execution before imagination can have a positive influence on the similarity of imagination and execution durations (Decety 1991; but see Rieger & Massen, 2014), we analyzed whether the order of action conditions mattered. Because there were no significant main effects or interactions with the factor order (imagination first, execution first), this factor is not included in the analyses reported here (see supplemental material for ANOVA results on order effects). Furthermore, we analyzed internal consistency of each task by using Cronbach’s alpha (see supplemental material). In addition, we calculated Pearson correlations within tasks (between IMA, EXE, and EXE actual) and between tasks (using the differences between IMA and EXE of IRIs, accuracy ratings averaged over tempi, and slopes).

## Results

### Number and duration task

#### Invalid button presses in execution

A repeated-measures ANOVA with the factors task (number task, duration task) and pattern (symmetric, parallel) was performed on the percentage of invalid button presses (number task, symmetric: 2.4%, parallel: 4.1%; duration task, symmetric: 1.8%, parallel: 2.3%). None of the effects were significant, all *Fs* ≤ 1.

#### Inter response intervals

**Fig. 3:**
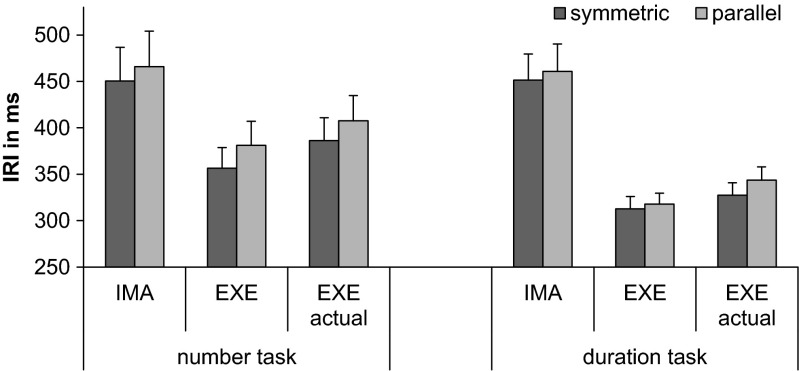
Means and standard errors of inter-response intervals (IRIs) in imagination (IMA), execution based on reported data (EXE), and execution based on actual performance (EXE actual) for symmetric and parallel movements in the number task and the duration task

Mean IRIs can be seen in Fig. 3. In a first step, a repeated-measures ANOVA with the factors task (number task, duration task), action (EXE, IMA), and pattern (symmetric, parallel) was performed on IRIs. A significant main effect of action, *F*(1, 20) = 25.3, *p* < .001, η^*2*^_*p*_ = .56, indicated longer IRIs in IMA (*M* = 457 ms) than in EXE (*M* = 342 ms). A significant main effect of pattern, *F*(1, 20) = 12.4, *p* = .002, η^*2*^_*p*_ = .38, indicated longer IRIs in parallel movements (*M* = 406 ms) than in symmetric movements (*M* = 393 ms). The significant interaction between task and action, *F*(1, 20) = 7.1, *p* = .015, η^*2*^_*p*_ = .26, indicated that in execution IRIs were significantly longer in the number task (369 ms) than in the duration task (315 ms; *p* = .026). In imagination IRIs did not significantly differ between tasks (number task: 458 ms; duration task: 456 ms; *p* = .94). None of the remaining effects were significant (task: *F*(1, 20) = 1.5, *p* = .24, η^*2*^_*p*_ = .07; task x pattern: *F*(1, 20) = 3.8, *p* = .066, η^*2*^_*p*_ = .16; action x pattern: *F* < 1; task x action x pattern: *F*(1, 20) = 1.6, *p* = .22, η^*2*^_*p*_ = .08).

To investigate further whether coordination constraints had a similar effect on imagination and execution, we conducted a control analyses. Differences between symmetric and parallel movements may depend on movement tempo, and movements were performed faster in execution than in imagination. We therefore calculated the percentage difference between coordination patterns as (IRI parallel - IRI symmetric) /IRI symmetric *100 separately for imagination and execution in each task. A repeated-measures ANOVA with the factors task (number task, duration task) and action (EXE, IMA) on the percentage difference in coordination pattern revealed no significant effects (task: *F*(1, 20) = 3, *p *= .1, η^*2*^_*p*_ = .13; action: *F*(1, 20) = 1.4, *p* = .26, η^*2*^_*p*_ = .06; task x action: *F*(1, 20) = 1.8, *p* = .2, η^*2*^_*p*_ = .08). This confirms that the coordination pattern did not significantly differ between tasks or between actions.

In a second step, a repeated-measures ANOVA with the factors task (number task, duration task), measure (actual, reported), and pattern (symmetric, parallel) was performed on IRIs. A significant main effect of task, *F*(1, 20) = 6.7, *p* = .018, η^*2*^_*p*_ = .25, indicated longer IRIs in the number task (*M* = 382 ms) than in the duration task (*M* = 325 ms). A significant main effect of measure, *F*(1, 20) = 64.5, *p* < .001, η^*2*^_*p*_ = .76, indicated that IRIs based on participants’ reports (*M* = 342 ms) were shorter than actual IRIs (*M* = 366 ms). The significant interaction between task and measure, *F*(1, 20) = 7.5, *p* = .013, η^*2*^_*p*_ = .27, indicated that the difference between actual IRIs and reported IRIs was significantly larger in the number task (28 ms) than in the duration task (20 ms; *p* = .013, *d* = .52). A significant main effect of pattern, *F*(1, 20) = 22.9, *p* < .001, η^*2*^_*p*_ = .53, indicated that IRIs were longer in parallel (*M* = 363 ms) than in symmetric movements (*M* = 346 ms). The interaction between task and pattern was significant, *F*(1, 20) = 5.4, *p* = .031, η^*2*^_*p*_ = .21, indicating that the difference between parallel and symmetric movements was significantly larger in the number task (23 ms) than in the duration task (11 ms; *p* = .031, *d* = .61). The remaining interactions were not significant (measure x pattern: *F*(1, 20) = 1.2, *p* = .29, η^*2*^_*p*_ = .06; task x measure x pattern: *F*(1, 20) = 3.2, *p* = .088, η^*2*^_*p*_ = .14.

### Synchronization task

#### Accuracy depending on speed

**Fig. 4:**
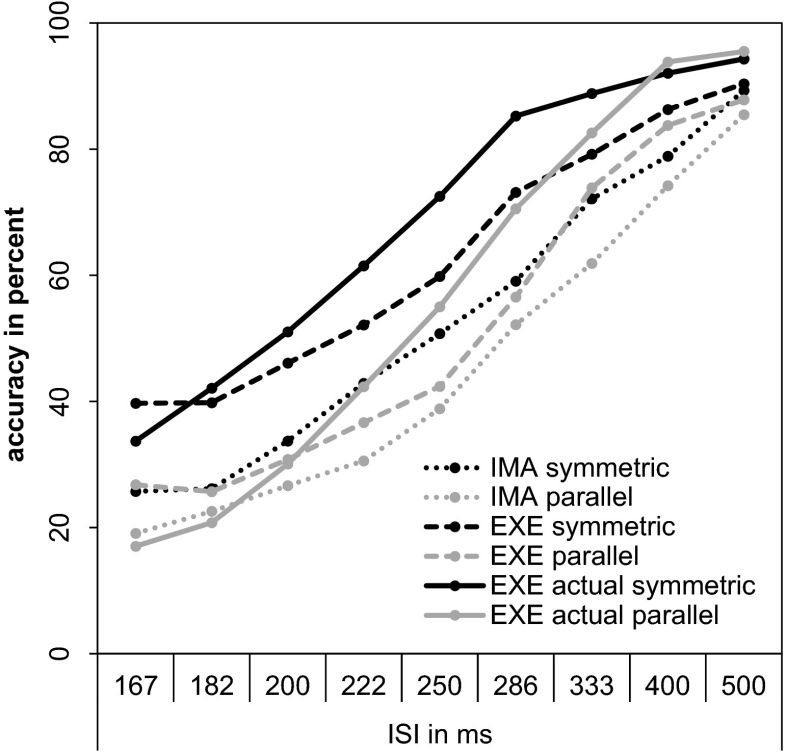
Mean percentages of correct responses in the synchronization task for symmetric and parallel movements in imagination (IMA), execution based on reported data (EXE), and execution based on actual performance (EXE actual) for the different inter-stimulus intervals (ISIs)

Mean reported and actual percentages of correct responses (COR%) can be seen in Fig. 4. In a first step, we compared COR% in IMA and EXE. A repeated-measures ANOVA with the factors action (EXE, IMA), pattern (symmetric, parallel), and ISI (167, 182, 200, 222, 250, 286, 333, 400, and 500 ms) was performed on COR%. A significant main effect of ISI, *F*(1.9, 38.4) = 90,9, *p* < .001, η^*2*^_*p*_ = .82, indicated that COR% decreased with faster tempi. A significant main effect of action, *F*(1, 20) = 16.1, *p* = .001, η^*2*^_*p*_ = .45, indicated lower COR% in IMA (*M* = 49%) than in EXE (*M* = 57%). A significant main effect of pattern, *F*(1, 20) = 41.2, *p* < .001, η^*2*^_*p*_ = .67, indicated lower COR% in parallel movements (*M*=49%) than in symmetric movements (*M*=58%). The significant interaction between action and pattern, *F*(1, 20) = 5.8, *p*= .026, η^*2*^_*p*_ = .22, indicated that the difference between parallel and symmetric movements was larger in EXE (mean difference = 11.4%) than in IMA (mean difference = 7.5%, *p* = .026). The interaction between pattern and ISI was significant, *F*(6.1, 133.9) = 3.8, *p* = .004, η^*2*^_*p*_ = .13, indicating that the difference between parallel and symmetric movements increased with increasing tempo. The difference between patterns in longer ISIs (400 ms and 500 ms) was significantly smaller than the difference in the shorter ISIs (167-250 ms; *p*_max_ = .025). The difference also was significantly smaller at ISI 400 than at ISI 286 (*p* = .006) and significantly smaller at ISI 333 than ISI 222 (*p* = .049). All remaining comparisons were not significant (*p*_min_ = .06). None of the remaining effects were significant (action x ISI: *F*(4.1, 282.5) = 1, *p* = .41, η^*2*^_*p*_ = .05; action x pattern x ISI: *F*(7.1, 143) = 2, *p* = .06, η^*2*^_*p*_ = .09).

In a second step we compared reported COR% and actual COR% in EXE. A repeated-measures ANOVA with the factors measure (actual, reported), pattern (symmetric, parallel), and ISI (167, 182, 200, 222, 250, 286, 333, 400, and 500 ms) was performed on COR%. A significant main effect of ISI, *F*(2.1, 42.1) = 125.6, *p* < .001, η^*2*^_*p*_ = .86, indicated lower COR% with fast tempi than with slower tempi. A significant main effect of pattern, *F*(1, 20) = 78.1, *p* < .001, η^*2*^_*p*_ = .8, was modified by the significant interaction between pattern and ISI, *F*(5.2, 104.2) = 11.4, *p* < .001, η^*2*^_*p*_ = .36. COR% was significantly higher in symmetric than parallel movements in fast tempi (ISI: 167-333 ms: *p*_max_ = .018) but not in slow tempi (400 and 500 ms: *p*_min_ = .39). The significant interaction between measure and ISI, *F*(2.1, 41.8) = 5.5, *p* = .007, η^*2*^_*p*_ = .22, indicated that actual COR% was significantly higher than reported COR% in slow tempi (250-500 ms: *p*_max_ = .032) but not in fast tempi (167-222 ms: *p*_min_ = .15). None of the remaining effects were significant (measure: *F*(1, 20) = 2.5, *p* = .13, η^*2*^_*p*_ = .11; measure x pattern: *F*(1, 20) = 1.1; *p* = .32, η^*2*^_*p*_ = .05; pattern x measure x ISI: *F*(6.1, 121.1) = 1.6, *p* = .16, η^*2*^_*p*_ = .07).

#### Slopes of logistic regressions (β_1_)

**Fig. 5:**
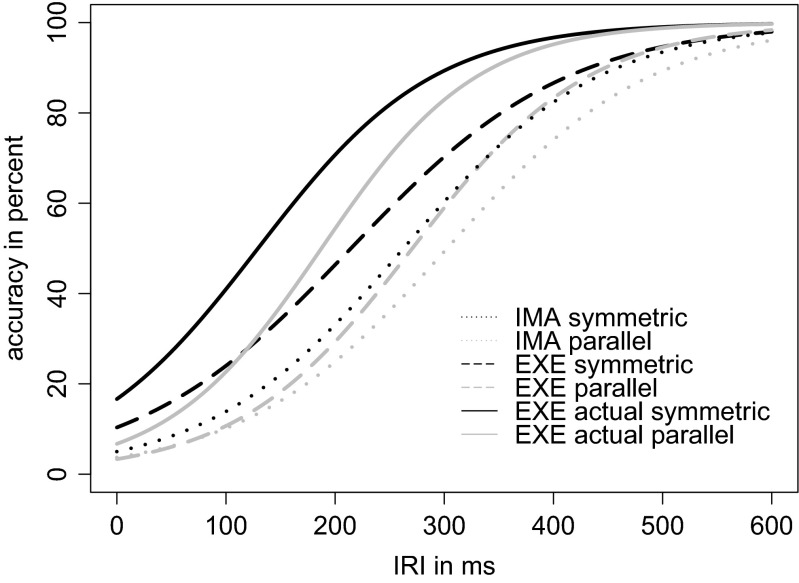
Mean logistic regression functions based on the data from the synchronization task for symmetric and parallel movements in imagination (IMA), execution based on reported data (EXE), and execution based on actual performance (EXE actual)

Averaged logistic regression functions can be seen in Fig. 5. In a first step, we compared the slopes in IMA and EXE. A repeated-measures ANOVA with the factors action (EXE, IMA) and pattern (symmetric, parallel) was performed on slopes. The significant interaction between action and pattern, *F*(1, 20) = 5.1, *p* = .035, η^*2*^_*p*_ = .2, indicated that slopes were steeper in parallel (*M* = 0.012) than in symmetric movements (*M* = 0.01) in execution (*p* = .024, *d* = .33) but not in imagination (parallel: *M* = 0.011; symmetric: *M* = 0.011; *p* = .4, *d* = .08). The main effects were not significant (action: *F* < 1; pattern: *F*(1, 20) = 4.2, *p* = .055, η^*2*^_*p*_ = .17).

In a second step, we compared participants’ reports and actual data in EXE. A repeated-measures ANOVA with the factors measure (actual, reported) and pattern (symmetric, parallel) was performed on slopes. A significant main effect of pattern, *F*(1, 20) = 7.7, *p* = .012, η^*2*^_*p*_ = .28, indicated less steep slopes in symmetric (*M* = 0.011) than in parallel movements (*M* = 0.013). The remaining effects were not significant (measure: *F*(1, 20) = 2.6, *p* = .12, η^*2*^_*p*_ = .12; measure x pattern: *F* < 1).

### Internal consistency of tasks

Cronbach’s alpha (α ) was analyzed separately for action conditions, coordination patterns and requested number of bimanual presses (number task: range 0.76-0.98), durations (duration task: range 0.68-0.95), and tempi (synchronization task: range 0.53-0.91). Because Cronbach’s alpha was based on three observations in the number and the duration task, we additionally calculated Cronbach’s alpha in the synchronization task based on the first three observations (range 0.37-0.86). Detailed tables of internal consistencies are shown in the supplemental material.

### Correlations within tasks

Pearson correlations within tasks were calculated separately for symmetric and parallel movements (Table 1). In all tasks reported performance in imagination (IMA) correlated significantly with reported performance in execution (EXE). Actual behavior (EXE actual) correlated significantly with reported performance in execution (EXE) in the number task and the duration task but not in the synchronization task.

**Table 1:** Pearson correlations within tasks separately for symmetric and parallel movements

	IMA x EXE		EXE x EXE actual		IMA x EXE actual	
	Symmetric	Parallel	Symmetric	Parallel	Symmetric	Parallel
Number task (IRI)	.68*	.68*	.99*	.995*	.7*	.71*
Duration task (IRI)	.61*	.68*	.92*	.95*	.73*	.69*
Synchronization task (accuracy scores)	.77*	.74*	.14	.42	.18	.55*
Regression slopes	.66*	.82*	.6*	.58*	.34	.58*

*p < 0.05 (critical *r* = 0.42)

IRIs and accuracy scores are based on reported performance in imagination (IMA) and execution (EXE) or on actual bimanual presses (EXE actual)

### Individual differences across tasks

To investigate whether individual differences in differences between imagination and execution are similarly reflected across tasks, we calculated the difference between imagination and execution for IRIs (number and duration task), accuracy ratings averaged over tempi, and slopes (synchronization task) and correlated them with each other. The correlation between the imagination-execution difference in IRI in the number task and the duration task was significant (*r* = .71). However, the imagination-execution differences in the synchronization task (accuracy and slopes) did not significantly correlate with the imagination-execution difference in IRI in the other tasks (−.23 < *r* < −.05). For correlations between tasks separately for the different measures (imagination, reported execution and actual execution) and the coordination patterns (symmetric and parallel), see supplemental material.

## Discussion

We investigated whether bimanual coordination constraints (symmetric movements are easier than parallel movements) are observed in motor imagery. Moreover, we were interested in whether the way of implementing the mental chronometry paradigm influences the presence of bimanual coordination constraints in motor imagery. Participants performed repetitive bimanual movements in three different tasks: the number task, the duration task, and the synchronization task. Results were similar across tasks. Both in imagination and execution, and in all three tasks, symmetric movements were easier to perform (shorter IRIs, higher COR%) than parallel movements. Furthermore, imagination and execution were correlated. Also differences were observed: imagination took longer than execution (number and duration tasks), imagination was less accurate than execution (synchronization task), the coordination constraint had a stronger influence on execution than on imagination (synchronization task only), and psychometric functions did not differ in steepness in imagination, but in execution the psychometric function was steeper for parallel than for symmetric movements (synchronization task).

### Bimanual coordination constraints in imagination and execution

The first goal of the present study was to investigate the functional equivalence of imagination and execution in repetitive bimanual movements. In imagination and execution (reported and actual) and in all three tasks, symmetric movements were easier to perform (shorter IRIs, higher COR%) than parallel movements. Furthermore, reported performance in imagination and execution was correlated in all tasks. These results are in line with previous research on bimanual coordination using repetitive bimanual movements, which demonstrate that participants are less accurate in parallel than in symmetric movements (Mechsner et al., 2001; Salesse, Oullier & Temprado, 2005) and that participants are less synchronous in parallel than in symmetric movements (Mechsner et al., 2001; Salesse et al., 2005). The observation that differences between coordination patterns were reduced with slower tempi (synchronization task) goes in line with previous results (Mechsner et al., 2001). We did not observe a disappearance of the symmetry benefit with very fast tempi (although it was reduced) as we had expected. Probably the fast tempi were not fast enough for all participants to result in a breakdown of coordinated performance.

The results also are in agreement with previous studies on motor imagery of bimanual coordination (Dahm & Rieger, 2016; Heuer et al., 1998). In contrast to those studies in which movements were imagined with one hand and executed with the other hand (Heuer et al., 1998) or in which reaching movements were used (Dahm & Rieger, 2016), we investigated the presence of bimanual constraints in motor imagery using tasks in which repetitive bimanual movements of both hands were imagined. We were able to show that in this situation, imagined and executed movements follow the same bimanual coordination constraints and thus are (for the most part) functionally equivalent.

However, functional equivalence was not complete: imagination took longer than execution (number and duration tasks), imagination was less accurate than execution (synchronization task), the coordination constraint had a stronger influence on execution than on imagination (synchronization task only), and psychometric functions were steeper for parallel than for symmetric movements in execution but not in imagination (synchronization task). Previous studies already have shown that imagination sometimes takes longer than execution when performing movements as fast as possible (Radulescu, Adam, Fischer & Pratt, 2010). One explanation might be that people focus more on details of a movement in imagination than in execution (Guillot et al., 2004). Another explanation is that motor imagery entails more processes than motor execution, because perceptual information has to be retrieved from memory to produce vivid representations (Frank, Land, Popp & Schack, 2014).

Interestingly, in the synchronization task the slopes for symmetric and parallel movements did not significantly differ in imagination but in execution the slopes were steeper in parallel than in symmetric movements. The steepness of the slope indicates how much movement accuracy depends on tempo. Parallel movements are more difficult than symmetric movements and therefore movement accuracy decreases more with faster movements—one factor that is not adequately represented in motor imagery. This agrees with studies that show a different impact of movement constraints on imagination and execution (Chabeauti et al., 2012; Cerritelli et al., 2000). For instance, even though both executed and imagined movements conform to Fitts’ Law, slopes of imagined movements are shallower than slopes of actual movements (Macuga, Papailiou & Frey, 2012).

### Versions of the mental chronometry paradigm

The second goal of this study was to investigate whether different versions of the mental chronometry paradigm yield comparable results. We used three different tasks: the number task, the duration task, and the synchronization task. The major finding that symmetric movements are easier to perform than parallel movements was observed in all tasks. Furthermore, all tasks also provided evidence for differences between imagination and execution as outlined above. Besides those similarities, there were differences between the tasks.

In execution, IRIs were longer in the number task than in the duration task. In the number task, participants had to remember the requested number, which was not the case in the duration task. This may have resulted in higher cognitive load. Another explanation is that participants put more effort into moving as fast as possible when they do not know in advance how much time they have to perform the movements than when know how many movements are requested.

In the synchronization task, the slope was steeper for parallel than for symmetric movements in execution but not in imagination. Similarly, the difference in accuracy between parallel and symmetric movements was larger in execution than imagination. This implicates a weaker influence of the coordination constraint in imagination than in execution, which was only found in the synchronization task. By calculating the percentage difference between coordination patterns in imagination and execution, we ruled out that this similarity in the number and the duration tasks was caused by differences in movement speed between imagination and execution.

The synchronization task provides additional information to the other tasks. Because tempo is under experimental control, the speed accuracy-tradeoff is taken into account, different tempi can be implemented, and psychometric functions can be calculated. Therefore imagination and execution performance are compared under similar conditions. This enabled us to show that bimanual coordination constraints depend on movement tempo (Mechsner et al. 2001)—both in imagination and execution—and that the slopes of the psychometric functions describing coordination performance as a function of speed were steeper in parallel movements than in symmetric movements in reported and actual execution but not in imagination. However, in the synchronization task internal consistencies seemed lower than in the number and the duration tasks. Hence, more trials may be needed to obtain reliable data, which enlarges the duration of data acquisition in the synchronization task.

To analyze further whether similar processes contribute to the different tasks, we correlated differences between imagination and execution of the different dependent variables between tasks. Differences between imagination and execution might reflect inadequate imagery or limited imagery skills (Guillot & Collet, 2005). The imagination-execution differences in the number and the duration task correlated significantly, indicating that a common factor contributed to them. The imagination-execution differences in the synchronization task did not significantly correlate with the imagination-execution differences in the other tasks. However, because different dependent variables were correlated between tasks, we cannot be sure whether different factors contributed to the differences across tasks or whether this is due to the use of different measures.

In the synchronization task, participants not only had to imagine movements but had to recognize movement errors. This might have been difficult in imagination, because it has been shown that performance errors are not spontaneously imagined. Even when participants are asked to pay attention to errors, they do not imagine as many errors as occur in actual performance (Rieger, Martinez & Wenke, 2011). It therefore may seem surprising that the discrepancy in accuracy ratings between imagination and execution in the synchronization task was not larger. It could be that accuracy ratings were based on the experienced difficulty of a specific tempo rather than on actual error experience. However, errors that are reported during imagination depend on the type of error (Rieger et al., 2011). In the present task, participants had to detect deviations from the requested tempo (i.e., the pacing sound) and deviations from the requested movement (i.e. hand synchrony, coordination pattern). These may be types of errors that can be detected during imagination.

### Reported and actual performance

Throughout the analysis, we compared imagination and execution based on reported performance. This was done because imagination in our tasks was based solely on participants’ reports (accuracy, number of movements, or having completed a certain number of movements). Actual and reported performance also may differ in execution. Indeed, this is what we observed in our study.

Reported IRIs in execution were shorter than actual IRIs (see above). In the synchronization task, actual accuracy was higher than reported accuracy in slow tempi (in which accuracy was very high), which might indicate a central tendency bias (Hollingworth, 1910). However, this was not confirmed by the analysis of the regression slopes. Between-participant variability was reflected in the correlations between reported and actual IRIs of the execution condition in the number and the duration task but not in the synchronization task. Nevertheless, correlations between reported measures in imagination and execution were significant in all tasks. One may argue that motor imagery is more closely related to the perceived performance than to the actual performance.

In all tasks, performance reports reflected coordination constraints that were apparent in actual performance. Thus, performance reports can be regarded as a valid operationalization of actual performance to investigate movement constraints.

### Implications

The developed synchronization task provides an alternative way to investigate motor imagery at least with repetitive movements. Even though it takes longer to obtain reliable data than with more common versions of the mental chronometry paradigm, i.e., the number and the duration tasks, the potential benefits may, depending on the research question and time available, outweigh this drawback. Its use eliminates any debate about a potential speed-accuracy tradeoff, because tempo is experimentally manipulated. This allows stricter experimental control about individual differences in movement speed. Potential individual limitations in imagery ability depending on tempo may be detected. Additionally, motor imagery of fast movements that are impossible to execute can be investigated. Furthermore, the synchronization task enables the calculation of psychometric functions, which provides the opportunity to analyze a different set of dependent variables, which potentially may be used to answer new questions about motor imagery.

From a theoretical viewpoint, motor imagery under time pressure may entail slightly different mechanisms than motor imagery without time constraints. To speculate, with very fast tempi representations in imagery might become less vivid than without time pressure. Different mechanisms in imagination and execution depending on available time may have far-reaching consequences for mental practice in the applied field. Experts in sports, music, and many other domains use mental practice to enhance their performance in situations under time pressure (Bernardi, De Buglio, Trimarchi, Chielli & Bricolo, 2013; Hall, Rodgers & Barr, 1990).

## Conclusions

Although imagination durations were longer than execution durations in the present study, imagination and execution correlated significantly and bimanual coordination constraints were similarly observed in imagination and execution. These findings strengthen the assumption that imagination and execution share similar processes (Jeannerod, 1995). Several options are available to implement a motor imagery task. Having participants rate their performance accuracy in the synchronization task rather than measuring durations has the advantage that movement speed can be experimentally manipulated. This has the potential to investigate a whole set of previously difficult to assess questions concerning motor imagery. However, the required task repetitions to obtain reliable data might be relatively high, which prolongs data collection.

## Electronic supplementary material

Below is the link to the electronic supplementary material.

ESM 1 (PDF 254 kb)

ESM 2 (MP4 5649 kb)
