# Supplementary material for: Is there symmetry in motor imagery? Exploring different versions of the mental chronometry paradigm
Source: Atten Percept Psychophys. 2016 May 12;78:1794–805. doi: 10.3758/s13414-016-1112-9 (PMC4972863; doi:10.3758/s13414-016-1112-9)
Supplement: Supplementary file 1 — (PDF 254 kb) [file 13414_2016_1112_MOESM1_ESM.pdf]

SUPPLEMENTAL MATERIAL

Is there symmetry in motor imagery? Exploring different versions of the mental chronometry paradigm

Stephan F. Dahm [1] & Martina Rieger [1]

[1] UMIT - University for Health Sciences Medical Informatics and Technology

Detailed analyses of inter response intervals (IRIs)

in the number task and the duration task

Number task

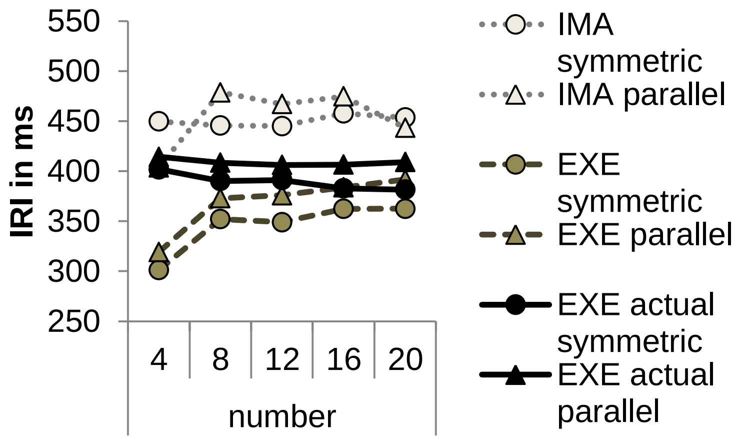

Figure SM1. Means and standard errors of inter response intervals (IRIs) in imagination (IMA), execution based on reported data (EXE), and execution based on actual performance (EXE actual) for symmetric and parallel movements, depending on requested number, in the number task

Duration task

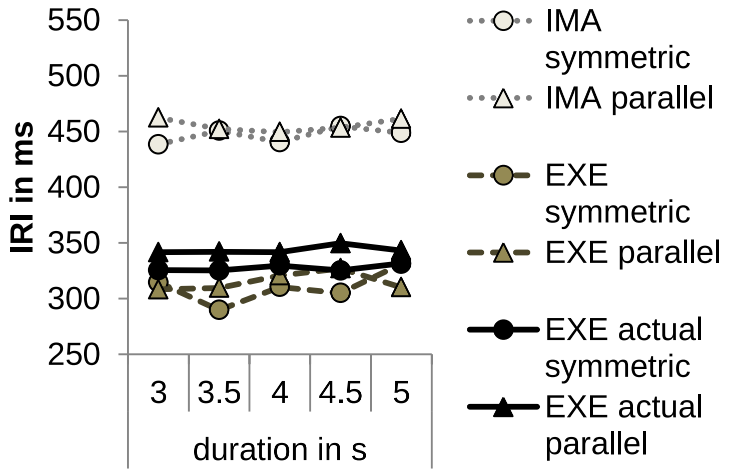

Figure SM2. Means and standard errors of inter response intervals (IRIs) in imagination (IMA), execution based on reported data (EXE), and execution based on actual performance (EXE actual) for symmetric and parallel movements, depending on requested duration, in the duration task.

Requested and actual bimanual button presses in the number task

Table SM1. *Mean number of requested, reported and actual bimanual presses in execution (standard deviation in parenthesis). Actual presses include invalid bimanual button presses.*

number task		duration task		
requested	actual	duration	reported	actual
		symmetric		
4	4.1 (0.5)	3 s	10.8 (2.1)	11.2 (2.1)
8	8.1 (0.4)	3.5 s	12.8 (2.4)	12.8 (2.6)
12	12.1 (1.0)	4 s	13.8 (2.9)	14.3 (2.8)
16	16.2 (1.3)	4.5 s	15.5 (2.6)	15.8 (2.9)
20	20.0 (1.0)	5 s	16.4 (3.4)	16.9 (3.7)
		parallel		
4	4.2 (0.7)	3 s	10.4 (1.7)	10.5 (2.0)
8	8.2 (0.8)	3.5 s	11.9 (2.1)	12.3 (2.1)
12	12.0 (0.4)	4 s	13.2 (2.4)	13.5 (2.6)
16	16.1 (0.6)	4.5 s	14.7 (3.0)	14.7 (3.0)
20	19.9 (0.8)	5 s	16.1 (2.8)	16.3 (2.9)

Correlations between tasks

Pearson correlations between the tasks, separately for the different measures (imagination, reported execution and actual execution) and the coordination patterns (symmetric and parallel) can be seen in Table SM2. Note that whereas correlations between the number and the duration task are positive (IRIs are correlated), they are negative between those tasks and the synchronization task (higher accuracy in the synchronization task coincides with shorter IRIs).

Table SM2. *Pearson correlations between tasks.*

imagination		reported execution		actual execution	
symmetric	parallel	symmetric	parallel	symmetric	parallel
IRI number task x IRI duration task					
.71*	.68*	.37	.40	.48*	.54*
IRI number task x COR% synchronization task					
-.31	-.16	-.15	.02	-.46*	-.34
IRI duration task x COR% synchronization task					
-.37	-.03	-.11	-.12	-.35	-.02

Reliability - internal consistency

Number task

Table SM3. *Cronbach's alpha for reported IRIs in the* ***number task.*** *In execution and imagination 3 observations are available to calculate internal consistency.*

requested number	execution		imagination	
	symmetric	parallel	symmetric	parallel
4	.14	.66	.61	.72
8	.91	.79	.94	.85
12	.76	.98	.91	.93
16	.94	.96	.97	.97
20	.97	.91	.97	.95

Duration task

Table SM4. *Cronbach's alpha for reported IRIs in the* ***duration task.*** *In execution and imagination 3 observations are available to calculate internal consistency.*

duration in s	execution		imagination	
	symmetric	parallel	symmetric	parallel
3	.88	.93	.89	.89
3.5	.92	.87	.90	.85
4	.92	.88	.95	.68
4.5	.88	.83	.86	.91
5	.93	.85	.93	.90

Synchronization task

Table SM5. *Cronbach's alpha for reported accuracies in the* ***synchronization task based on 5 accuracy estimates.***

		execution		imagination	
ISI (ms)	tempo (bpm)	symmetric	parallel	symmetric	parallel
500	120	.89	.88	.88	.90
400	150	.87	.91	.84	.83
333	180	.81	.91	.84	.88
286	210	.81	.82	.84	.84
250	240	.76	.76	.80	.77
222	270	.75	.83	.77	.58
200	300	.77	.63	.71	.62
184	330	.80	.82	.80	.53
167	360	.74	.68	.81	.80

Table SM6. *Cronbach's alpha for reported accuracies in the* ***synchronization task*** *based on the first three repetitions.*

		execution		imagination	
ISI (ms)	tempo (bpm)	symmetric	parallel	symmetric	parallel
500	120	.81	.71	.82	.76
400	150	.80	.83	.76	.72
333	180	.59	.86	.67	.72
286	210	.63	.81	.74	.75
250	240	.69	.76	.66	.62
222	270	.75	.73	.68	.37
200	300	.74	.61	.61	.43
184	330	.81	.71	.62	.40
167	360	.36	.68	.72	.72

Simultaneousness of hands (inter onset intervals, IOIs)

For all tasks we calculated the inter onset interval (IOI), i.e. the absolute value of the interval between button presses of the left and the right hand of valid bimanual presses as a measure of simultaneousness. Means and standard errors of IOIs can be seen in Figure SM3. A repeated measures ANOVA with the factors pattern (symmetric, parallel) and task (number task, duration task, synchronization task) was performed on IOIs. A significant main effect of pattern, $F(1, 20)=72.7$, $p<.001$, $\eta^2_p=.78$, indicated shorter IOIs in symmetric (M=13 ms) than in parallel movements (M=19 ms). The significant interaction between pattern and task, $F(2, 40)=3.7$, $p=.034$, $\eta^2_p=.16$, indicated that the difference between IOIs of parallel and symmetric movements was significantly smaller in the number task (4.6 ms) than in the duration task (7.7 ms; $p=.023$, $d=.63$). The difference in the synchronization task (7.7 ms) did not differ significantly from the differences in the other tasks ($p_{min}=.079$). The main effect task was not significant, $F<1$. Thus, in all three tasks, IOS were lower in symmetric movements (higher simultaneousness) than in parallel movements. This further supports the observation that symmetric movements are easier to perform than parallel movements.

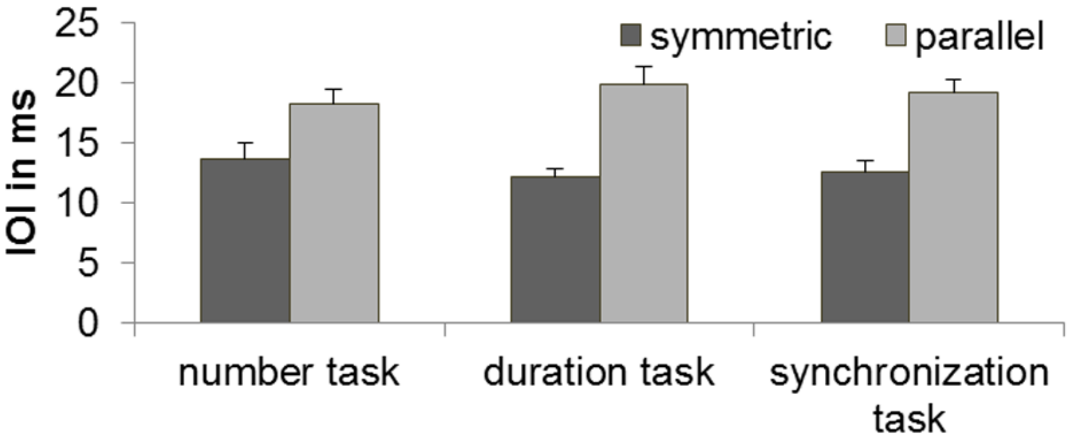

Figure SM3. Means and standard errors of inter onset intervals (IOIs) for symmetric and parallel movements in the number task, the duration task, and the synchronization task.

Strength of representation

In addition to performance, we investigated the strength of kinesthetic/tactile and visual representation during imagination and execution using performance reports. This was of interest, because even when imagination and execution durations are similar, movements might still be represented in a slightly different way. Given the equivalence assumption (Jeannerod, 1995), no differences between imagination and execution in the strength of kinesthetic/tactile and visual representations should be observed. However, previous results indicate that kinesthesis/touch might be less strongly represented in imagination than in execution when performing bimanual reaching movements (Dahm & Rieger, 2016).

Means and standard errors of the strength of representation can be seen in Figure SM4. A repeated measures ANOVA with the factors task (number task, duration task, synchronization task), action (EXE, IMA) and modality (kinesthesis/touch, vision) was performed on strength of representation. A significant main effect of modality, $F(1, 20)=8.7$, $p=.008$, $\eta^2_p=.3$, indicated stronger kinesthetic/tactile ($M=75$) than visual representations ($M=62$). None of the remaining effects was significant (task: $F<1$; action: $F(1, 20)=1.7$, $p=.21$, $\eta^2_p=.08$; task x action: $F(1.7, 34.4)=2.5$, $p=.1$, $\eta^2_p=.11$; task x modality: $F<1$; action x modality: $F(1, 20)=1.2$, $p=.28$, $\eta^2_p=.06$; task x action x modality: $F(2, 39)=2.1$, $p=.14$, $\eta^2_p=.09$;).

In line with previous results (Dahm & Rieger, 2016) execution and imagination did not differ significantly from each other and stronger representation of kinesthesis/touch than vision was observed. Importantly, these effects did not significantly differ between tasks and the the strength of representation did not significantly differ between imagination and execution.

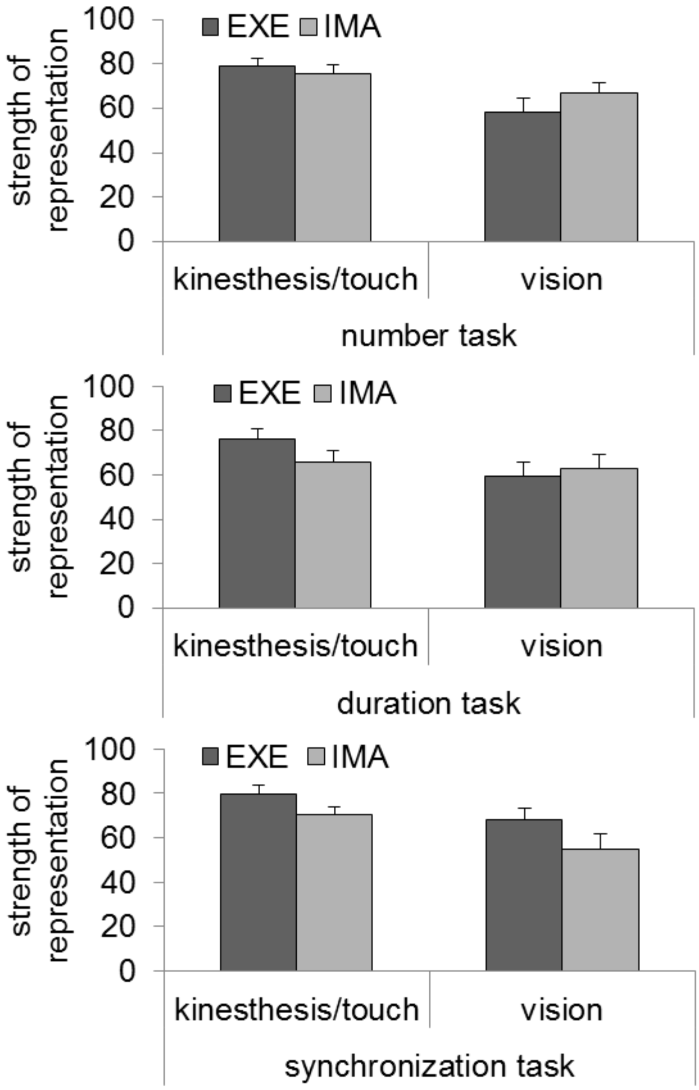

Figure SM4. Means and standard errors of the strength of kinesthetic/tactile and visual representations in execution (EXE) and imagination (IMA) in the number task, the duration task, and the synchronization task.

Order effects

For the number and duration task we calculated a repeated measures ANOVA with the between factor order (EXE first, IMA first) and the within-factors task (number, duration), action (EXE, IMA), and pattern (symmetric, parallel) on IRI. For the synchronization task we calculated a repeated measures ANOVA with the between factor order (EXE first, IMA first) and the within-factors action (EXE, IMA), pattern (symmetric, parallel), and ISI (167, 182, 200, 222, 250, 286, 333, 400, and 500 ms) on COR%. Neither the main effects of action order nor any of the interactions with order became significant in both analyses. The results of the ANOVAs can be seen in Table SM7.

Table SM7. *ANOVA results of the analyses of order effects.*

Number and duration tasks				
	F	*df1, df2*	*p*	η^2_p
order	1.6	1, 19	.23	.08
order x action	0.8	1, 19	.4	.04
order x task	0.2	1, 19	.65	.01
order x pattern	0.1	1, 19	.8	<.01
order x action x task	0.2	1, 19	.67	.01
order x action x pattern	0.3	1, 19	.62	.01
order x task x pattern	0.2	1, 19	.68	.01
order x action x task x pattern	0.3	1, 19	.57	.02
Synchronization task				
order	<0.1	1, 19	.91	<.01
order x action	<0.1	1, 19	.99	<0.1
order x ISI	0.7	1, 19	.51	.04
order x pattern	<0.1	1, 19	.9	<0.1
order x action x ISI	1.6	1, 19	.18	.08
order x action x pattern	<0.1	1, 19	.96	<0.1
order x ISI x pattern	.2	1, 19	.99	.01
order x action x ISI x pattern	1.2	1, 19	.29	.06
